# Segregation of the Anodic Microbial Communities in a Microbial Fuel Cell Cascade

**DOI:** 10.3389/fmicb.2016.00699

**Published:** 2016-05-11

**Authors:** Douglas M. Hodgson, Ann Smith, Sonal Dahale, James P. Stratford, Jia V. Li, André Grüning, Michael E. Bushell, Julian R. Marchesi, C. Avignone Rossa

**Affiliations:** ^1^Department of Microbial and Cellular Sciences, University of SurreyGuildford, UK; ^2^Cardiff School of Biosciences, Cardiff UniversityCardiff, UK; ^3^Warwick Integrative Synthetic Biology Centre, University of WarwickCoventry, UK; ^4^Centre for Digestive and Gut Health, Department of Surgery and Cancer, Imperial College LondonLondon, UK; ^5^Division of Computational and Systems Medicine, Department of Surgery and Cancer, Imperial College LondonLondon, UK; ^6^Department of Computer Science, University of SurreyGuildford, UK

**Keywords:** microbialfuel cells, microbial communities, electroactive bacteria, metagenomic analysis, metabolite profiling, anodic biofilms

## Abstract

Metabolic interactions within microbial communities are essential for the efficient degradation of complex organic compounds, and underpin natural phenomena driven by microorganisms, such as the recycling of carbon-, nitrogen-, and sulfur-containing molecules. These metabolic interactions ultimately determine the function, activity and stability of the community, and therefore their understanding would be essential to steer processes where microbial communities are involved. This is exploited in the design of microbial fuel cells (MFCs), bioelectrochemical devices that convert the chemical energy present in substrates into electrical energy through the metabolic activity of microorganisms, either single species or communities. In this work, we analyzed the evolution of the microbial community structure in a cascade of MFCs inoculated with an anaerobic microbial community and continuously fed with a complex medium. The analysis of the composition of the anodic communities revealed the establishment of different communities in the anodes of the hydraulically connected MFCs, with a decrease in the abundance of fermentative taxa and a concurrent increase in respiratory taxa along the cascade. The analysis of the metabolites in the anodic suspension showed a metabolic shift between the first and last MFC, confirming the segregation of the anodic communities. Those results suggest a metabolic interaction mechanism between the predominant fermentative bacteria at the first stages of the cascade and the anaerobic respiratory electrogenic population in the latter stages, which is reflected in the observed increase in power output. We show that our experimental system represents an ideal platform for optimization of processes where the degradation of complex substrates is involved, as well as a potential tool for the study of metabolic interactions in complex microbial communities.

## Introduction

The diverse microbial species present in natural environments interact with each other through metabolic and functional relationships that ensure the activity and stability of the community. Growth and survival of the species in the community depend on the exchange of metabolic products, especially in processes such as the degradation of complex natural polymers, such as polysaccharides, proteins, nucleic acids, and lipids (Sieber et al., 2012; Morris et al., 2013). Those synergistic interactions are the basis of natural processes such as the degradation of plant or animal residues, or the biogeochemical cycling of carbon, nitrogen or sulfur, but can also be essential in managed or artificial processes for agriculture, the food industry, wastewater treatment, or industrial bioprocesses (Brenner et al., 2008; Fuhrman, 2009; Chiu et al., 2014; Jacobsen and Hjelmsø, 2014).

The interactions among the species in a microbial consortium affect the function, activity, and stability of the community, providing improved metabolic capabilities. This is exploited in microbial fuel cells (MFCs), bioelectrochemical devices that convert organic or inorganic substrate chemical energy into electrical energy, by the metabolic activity of microorganisms. The power output of an MFC will depend on how efficiently the anodic biofilm catalyzes the decomposition of the fuel source and transfers electrons to the anode (Wrighton et al., 2010). While single microbial species are very efficient for the conversion of simple molecules into electricity, MFCs inoculated with microbial communities are used to catalyze the degradation of substrate mixtures (Feng et al., 2008; Greenman et al., 2009). Efficient degradation of complex feedstocks requires the complete breakdown of different macromolecules, achievable only through the combination of diverse microbial metabolic activities and long residence times in the anode chamber.

In an MFC inoculated with a natural microbial community, acclimatization to a given substrate drives the assembly of anode-associated mixed species biofilms toward definable consortia (Chae et al., 2009; Yates et al., 2012). The anaerobic environment in the MFC anode chamber results in a microbial community composed predominantly of fermentative bacteria and electrogenic anodophilic species. Our recent studies have demonstrated that increased power output is associated with both elevated microbial diversity (Stratford et al., 2014) and abundance of anaerobic respirators (Grüning et al., 2015). Fermentative species are unable to fully oxidize carbohydrates and instead undergo fermentative metabolism (Pfeiffer et al., 2001), while electrogenic bacteria oxidize non-fermentable substrates (e.g., acetate), and transfer the resulting electrons to an electron acceptor (EA; Kiely et al., 2011; Sun et al., 2015). Previous reports in single MFCs have suggested a syntrophic link between fermentation and electrogenesis (Freguia et al., 2008; Kimura and Okabe, 2013), but no assignment of specific taxa participating in the relationship has been provided. Syntrophic interactions have been studied using single substrates in MFCs (Lu et al., 2012) or in microbial electrolysis cells (MECs), a different bioelectrochemical system (Sun et al., 2012), while metabolic interactions and the mechanisms of electron transfer have been analyzed in binary systems, confirming syntrophy in co-cultures of two species (Butler et al., 2009; Rotaru et al., 2012).

Although almost all of the studies reported make use of individual MFCs, systems where a series of smaller MFCs units is connected in a hydraulic cascade (“stack”) have resulted in improved power output and increased efficiency (Gálvez et al., 2009; Gurung and Oh, 2012; Winfield et al., 2012; Zhuang et al., 2012; Ledezma et al., 2013). Therefore understanding the microbial ecology of such cascading systems is key to improving their yield and stability. In this work, we studied the anodic microbial communities and their associated metabolism in a cascade of MFCs fed with dried distiller’s grain with solubles (DDGS), a downstream product of the bioethanol industry (Eskicioglu et al., 2011) which has not been previously reported as a feed substrate in MFCs. We analyzed the changes in the taxonomic, metabolic, and electrochemical characteristics of each MFC within the cascade, with the objective of understanding the functional and structural modifications of the microbial community involved in the process. We show that communities with different metabolic characteristics can be identified along the cascade, as a result of the metabolic interactions between species. We suggest that the experimental approach presented would be applicable to other processes involving microbial communities.

## Materials and Methods

### Preparation of DDGS Medium

The medium used throughout this study was 10% w/v DDGS, prepared by autoclaving a 10% (w/v) suspension of wheat DDGS in distilled water for 1 h at 121°C. The DDGS slurry obtained was then sieved through a 0.3 mm mesh (Endecotts Ltd., UK) and centrifuged at 6370× *g* for 30 min to remove insoluble particles. The medium was adjusted to pH 7 and autoclaved at 121°C for 15 min. The composition of the 10% DDGS medium was (g.l^-1^): total carbohydrates (as glucose equivalents), 16.80; free glucose, 0.14; glycerol, 6.60; pentosans, 2.98; L-lactate, 1.61; total phosphates, 0.67.

### Preparation of MFC Inoculum

A microbial community derived from lignocellulose compost was used as the inoculum for the MFC cascade. The community was obtained by vigorously mixing 10% w/v lignocellulosic compost with PBS and inoculating the liquid fraction into a bioreactor (working volume 1 l), which was continuously fed with 10% DDGS medium (flow rate = 50 ml.h^-1^) for 840 h. The enrichment process was performed under aerobic and anaerobic conditions, and both enriched communities were tested for their electrogenic activity in single MFCs. The aerobically enriched community generated a higher peak power at 120 h, and was therefore chosen as the inoculum for the MFCs used in this study (**Supplementary Figure S1**).

### MFCs Design and Operation

The single-chamber MFCs used in this study were designed to allow anode biofilm samples to be removed without the need to disassemble the MFC. Each cell consisted of a 140 cm^3^ Perspex anode chamber with Perspex plates on either side. Air was able to access the cathode through four 0.5 cm × 4 cm slots cut into the cathode-side plate. Insulated Ni/Cr wire (Advent Research Materials, UK) was threaded through the cathode and protruded between the plate and chamber. The anode consisted of an 8 cm by 22 cm carbon fiber cloth wrapped around a Perspex rod, with insulated Ni/Cr wire threaded through and around the carbon fiber cloth and rod; held in place with a rubber bung. The exposed anode surface area was 96.5 cm^2^.

The air-breathing cathode consisted of 410 μm thick carbon cloth, coated with 4 mg cm^2^ of Pt black catalyst with polytetrafluoroethylene binder (FuelCellsEtc, USA) and was hot-pressed onto Nafion^®^ 115 proton-exchange membrane (DuPont, USA) as previously described (Beecroft et al., 2012).

The working volume of each MFC was 127 cm^3^. A magnetic bar placed in each anode compartment was used to ensure sufficient mixing of the anolyte suspension.

### MFC Cascade

**FIGURE 1:**
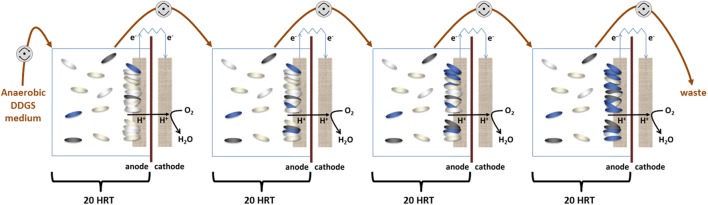
**Schematic representation of the MFC cascade setup**. Four identical MFCs are connected hydraulically by oxygen impermeable tubing (brown arrows). Fresh medium is pumped into the first MFC, and then cascaded sequentially through each cell driven by peristaltic pumps (gray circles) ensuring a hydraulic retention time (HRT) of 20 h for each cell. The oval shapes represent the species in the planktonic and biofilm populations in the anode chamber.

Four MFCs were connected hydraulically, with the eﬄuent of one MFC feeding into the next downstream (**Figure 1**). The medium was continuously purged with oxygen-free nitrogen gas (OFN) and supplied to the MFC cascade at a flow rate of 6.35 ml.h^-1^, resulting in a hydraulic retention time (HRT) of 20 h for each MFC in the cascade (80 h for the whole cascade). Each MFC was inoculated with 1 ml of enriched lignocellulosic culture and operated in batch mode for the first 24 h. The MFCs were operated at 30°C and samples taken for microbial and chemical analysis every 120 h (six anode chamber volume changes) thereafter. All the results were obtained from three independent biological replicates.

### Electrochemical Measurements

Microbial fuel cell voltage was monitored using an Arbin BT2143 battery tester controlled with MITS Pro software (Arbin Instruments, USA) across a fixed external resistance of 1000 Ω. The electrical current was calculated using Ohm’s law, *I* = *V*/*R*, where *V* is the measured voltage and *R* is the external resistance. Electrical power production was determined using the derivation of Joule’s law, where power *P* = *V* × *I*. Volumetric power and current density were calculated by dividing the output by the total anode chamber volume. Polarization curves were carried out for the single MFC units in the cascade every 120 h by connecting the MFC to external load values ranging from 700 000 to 250 Ω for 5 min intervals at each resistance, to ensure the MFC reached a stable output.

### Microbial Community Analysis

Total DNA was extracted from either the anode biofilm or the anolyte suspension using FastDNA Spin Kit for Soil (MP Biomedicals, UK). To sample the anodic biofilm, the anode, wrapped around a central Perspex rod, was removed briefly from the MFC set up in an aseptic environment. Biofilm samples were taken from the anode using a sterile scalpel and suspended in 1 ml of PBS containing 20% (w/v) glycerol and stored at -20°C. The anolyte suspension was sampled by taking 500 μl of the suspension under aseptic conditions and added to 500 μl of 40% (w/v) glycerol – PBS and stored at -20°C. Prior to DNA extraction, the samples were centrifuged (10 000 × *g*, 5 min), washed three times with 1 mL PBS and resuspended in 100 μl of nuclease free water (Promega, UK).

PCR and subsequent sequencing are described in the literature (Dowd et al., 2008) and were performed at the Research and Testing Laboratory (Lubbock, USA). The V1–V3 hypervariable regions of 16S rRNA genes were amplified for sequencing using the following forward and reverse fusion primers: 28F-GAGTTTGATCNTGGCTCAG and 519R-GTNTTACNGCGGCKGCTG. Trace data was deposited at the EMBL-EBI European Nucleotide Archive with the project accession PRJEB9971. Analysis of the 16S rRNA sequencing data was performed using Mothur v1.32.1 to v1.34.1 as previously described (MacIntyre et al., 2015). All OTUs were defined using a cut off value of 97%. Taxonomic relative abundances are available in the Supplementary Information.

### Chemical Analyses

All liquid samples (anolyte suspension or fresh medium) were filtered through 0.22 μm membranes (Millex, Merck Millipore Ltd., Ireland). Total carbohydrate analysis was performed using a colorimetric phenol/sulphuric acid method (Dubois et al., 1956). Total phosphate concentration was determined using a phosphate assay kit (Merck, Germany). L-lactate and glycerol were determined using EnzyChrom enzymatic assay kits ECLC-100 and EGLY-100, respectively (BioAssay Systems, USA). The concentrations of glucose and iron were determined using Sigma assay kits GAGO20 and MAK025 (Sigma–Aldrich, USA). The pentosan content in the medium was quantified using a colorimetric method (Finnie et al., 2006) and xylose concentration was measured using an enzymatic assay kit (Megazyme, Ireland). Acetate, succinate, and propionate in the suspension were determined using ^1^H NMR spectroscopy as previously described Li et al. (2011). Measurement of pH was performed on 10 ml of anolyte suspension using a pH-meter (Mettler Toledo MP220, Switzerland).

### Statistical Analysis

Linear mixed effect models were built wherein biological replicate (cascade run: 1,2,3) was included as a random factor, while fixed effects included time, fermenter abundance, and fermentation products. The significance associated with including a variable was determined by stepwise addition followed by likelihood ratio testing to compare the new model with the previous (null) model lacking the additional variable. Each model was assembled and tested in the order: intercept, time, abundance of fermenters, *or* metabolite concentration. For each model, standardized coefficients (β) and significances were calculated for effects associated with each independent variable.

Three models were constructed. Model (1) tested the hypothesis that time and hydraulic series order had distinct effects on MFC power output. This model also included an interaction between hydraulic series position and time. The interaction term was included to determine if the passage of time significantly altered the order of the hydraulic series. Models (2) and (3) were subsequently assembled to test the hypothesis that the concentration of acetate and fermenter abundance, respectively, predict power output. All statistical analysis was carried out using the LME4 package in R version 2.15.2

## Results

### Electrogenic Activity and Power Output of the MFC Cascade

The microbial community in the anode of an MFC converts the chemical energy of substrates into electricity through the metabolic activity of the species present. A direct evaluation of the electrogenic activity of the system can be obtained by measuring the power output. In our experimental system (Schematically represented in **Figure 1**), four individual MFCs were connected hydraulically and the voltage and power output were monitored during the experiment.

**FIGURE 2:**
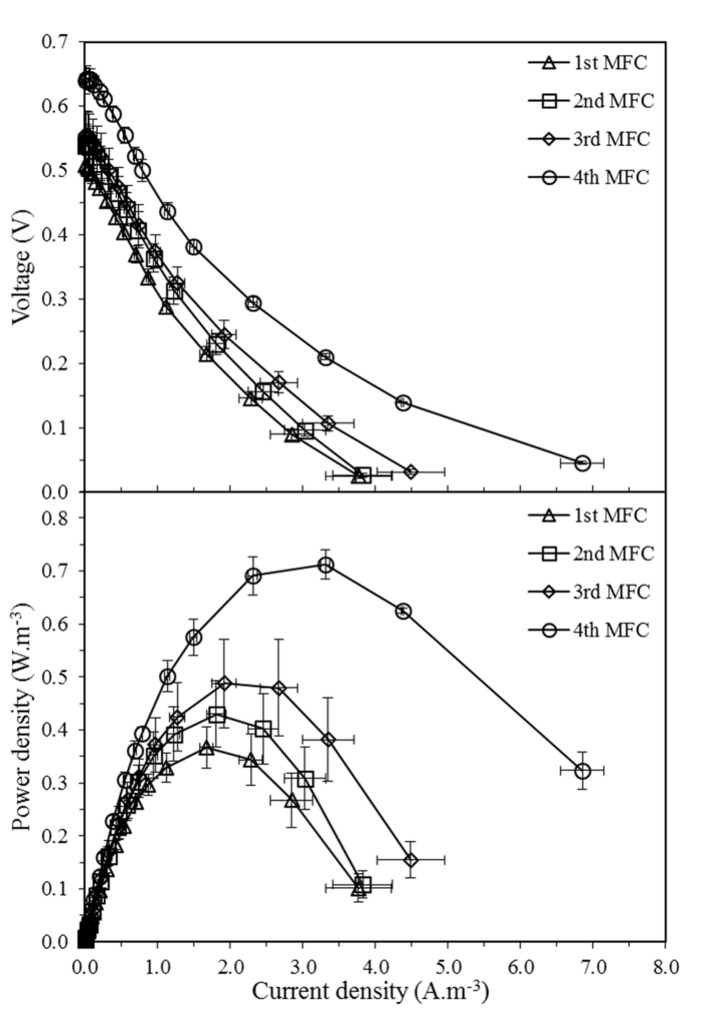
**Voltage output and volumetric power density curves as a function of volumetric current density of four MFCs connected hydraulically in cascade**. Results are mean values of three cascade biological replicates at 120, 240, 360, and 480 h. Error bars represent standard error of the biological replicates.

The mean maximum voltage observed in the first, second, third, and fourth MFCs were 0.51, 0.54, 0.55, and 0.64 V, respectively, while the mean peak powers attained were 0.37, 0.43, 0.50, and 0.73 W.m^-3^. The polarization curves (**Figure 2**) demonstrate that the medium fed at the selected flow rate facilitated power production in the four MFCs, with the performance of each MFC consistently increasing along the cascade.

In order to confirm that the increase of power along the MFC cascade is significant, a linear effects model was constructed to correlate power output with the time at which peak power was measured and with the position of the MFC in the cascade (**Table 1**, Model 1). The position of the MFC in the cascade showed a statistically significant positive correlation with peak power (β = 0.73, *p* ≤ 0.0001), confirming that the power output does indeed increase along the cascade. On the other hand, time had no significant effect on the relationship between the position of the MFC in the cascade and the peak power output (β = -0.21, *p* = 0.45), thus confirming that the power output of the MFCs in the cascade remained at steady state over the course of the experiment.

**Table 1:** Mixed effect models, testing the effect of cascade MFC position, time, fermentative population abundance and fermentation on peak power, assessed by β weight.

	Independent variables	β	*SE*	χ^2^	*p*
Model 1	Hydraulic series position	0.73	0.21	21	<0.0001^∗^
	Time	-0.36	0.21	26	0.0001^∗^
	Time^∗^series position	-0.21	0.29	0.6	0.45
Model 2	Acetate	0.67	0.15	16	<0.0001^∗^
	Time	-0.73	0.11	14	0.0001^∗^
Model 3	Total fermentative population	0.50	0.11	18	<0.0001^∗^
	Time	-0.44	0.10	15	0.0001^∗^

*^∗^Statistical significance value <0.05*.

### Metabolic Product Analysis

The concentrations of carbon sources and metabolic end products in the anolyte suspension were measured in each MFC during the experiment to obtain an indication of the activity of the different metabolic pathways in the species present in the communities. Identification of those pathways is essential to understand the metabolic mechanisms prevailing in each stage of the cascade.

**FIGURE 3:**
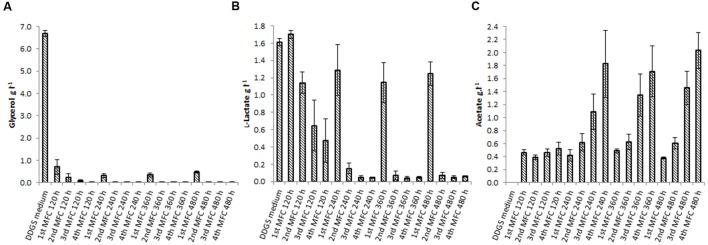
**Concentration of the fermentable carbohydrates glycerol **(A)** and lactate **(B)**, and the fermentation end product acetate **(C)** measured in the DDGS medium and the anolyte suspension across the MFC cascade**. Error bars are standard error of three cascade biological replicates, apart from the DDGS medium which are standard error of three technical replicates.

Dried distiller’s grain with solubles contains a variety of carbohydrates and other organic compounds, including glycerol, pentosans and glucose, that can be fermented by several of the species present in the community to generate short chain fatty acids (SCFAs) such as propionate, succinate, or acetate. In general, SCFAs are ideal carbon sources for anodophilic bacteria (Jang et al., 2010), with acetate being the preferred substrate for electron donation to the anode by various species (Choi et al., 2011; Sun et al., 2015). In the first MFC, glycerol (present in the feed at 6.60 g.l^-1^) was almost completely consumed (**Figure 3A**). Almost no lactate was consumed in that MFC (lactate concentration remained close to 1.6 g.l^-1^, the concentration in DDGS), but it was undetectable in the second MFC. Acetate concentration increased consistently across the cascade (**Figure 3C**), reaching a maximum concentration of 2.57 g.l^-1^. The concentration of acetate significantly correlates with peak power (β = 0.67, *p* ≤ 0.0001), as shown from the results of a linear effects model correlating power output with substrate concentration across the cascade (**Table 1**, Model 2).

Another significant source of organic carbon in the medium is the pentosan fraction, present at a concentration of ∼3 g.l^-1^. Although no free xylose was detected in DDGS, this sugar is most likely released by microbial hydrolysis of the pentosan fraction. It is present in the MFC cascade at the relatively low concentration of 0.20 g.l^-1^ (Supplementary Information). It has been reported that xylose in MFCs can be fermented into acetate (Huang and Logan, 2008; Mäkinen et al., 2013), which is used as a substrate for electricity production.

**FIGURE 4:**
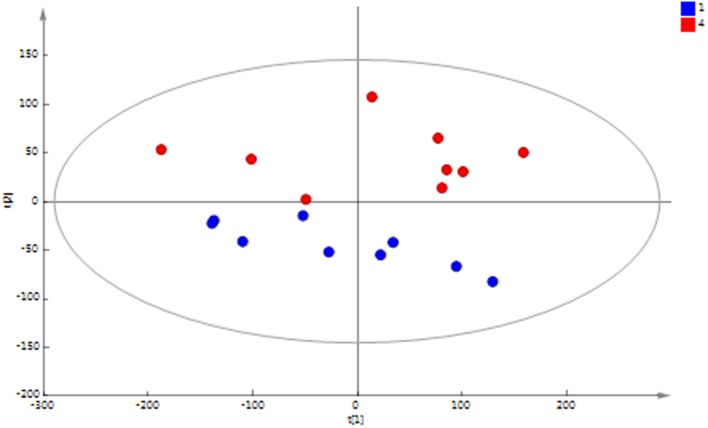
**PCA scores plot of ^1^H NMR spectral data of anolyte from MFC1 (blue) and MFC4 (red)**. A clear separation between the initial and final MFCs in the cascade along the second principal component (PC2) is observed [*R*^2^*X* (PC1) = 57.2%; *R*^2^*X*(PC2) = 14.5%; *Q*^2^= 0.53].

**FIGURE 5:**
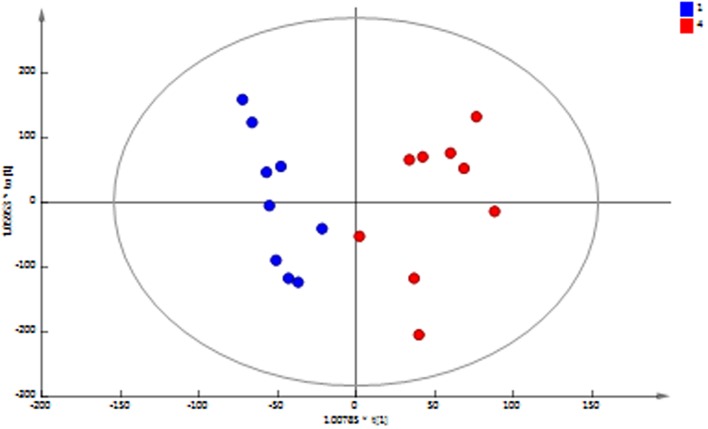
**OPLS-DA scores plot of ^1^H NMR spectral data of anolyte from MFC1 (blue) and MFC4 (red) shows a significant difference between the initial and final MFCs in the cascade (*R*^2^*X* = 71.5%; *Q*^2^*Y* = 0.78; cross-validated ANOVA *p* = 0.0003)**.

Principal component analysis (PCA) of the ^1^H NMR spectral data of the MFC cascade anolyte suspension (**Figure 4**) shows a clear metabolic shift between the first and last MFC in the cascade along the second principal component, which is further supported by a supervised orthogonal partial least squares discriminant analysis (O-PLS-DA) between the two groups (**Figure 5**). These results strongly suggest a segregation of the anodic communities across the cascade.

### Microbial Community Analysis

The observed changes in the power output and in the metabolite profile of the MFC cascade are suggestive of changes in the metabolic activity of the anodic microbial community. The composition, dynamics, and taxonomy of the bacterial communities in the anodic biofilm and in the anolyte suspended culture were analyzed by next-generation sequencing of the amplified 16S rRNA genes of the entire microbial community in each sample. To avoid PCR-induced artifacts and bias (Youssef et al., 2009; Engelbrektson et al., 2010), the same DNA extraction, amplification, and sequencing methods were applied to all samples to allow fair comparison of taxonomic data sets (Dowd et al., 2008; MacIntyre et al., 2015).

Four genera made up on average 74.3% of the microbial communities observed in each MFC anodic biofilm and anolyte suspension. In order of mean % relative abundance, these were: *Clostridium* (35.7±14.1%), *Rummeliibacillus* (19.0±5.9 %), *Lactococcus* (11.4±3.5 %), and *Bacteroides* (8.2±3.6%). Ten other genera with lower abundancies were *Streptococcus* (4.1±1.8%), *Enterococcus* (3.9±1.3%), *Proteiniclasticum* (3.3±2.7%), *Dysgo nomonas* (1.1±0.3%), and *Leuconostoc* (1.1±0.3%), *Ethanoligenes* (0.8±0.4%), *Comamonas* (0.7±0.2%), *Sporobacterium* (0.7±0.5%), *Anaerotruncus* (0.6±0.4%), and *Stenotrophomonas* (0.5±0.2%). Those 14 strains made up to 96.1% of the identified genera in the population. The complete list of identified genera and their relative abundances are available in the Supplementary Information.

Each genus was assigned a metabolic class with respect to terminal EAs according to their typical metabolism according to the literature (**Table 2**). These were either fermenters, which use intracellular metabolites as EAs, or anaerobic respirators, which are able to fully oxidize fermentation products using a terminal EA other than oxygen, such as sulfate, nitrate or an external anode with the appropriate redox potential. However, some species are capable of performing either metabolic function, depending on the prevalent environmental and/or physiological conditions. As it is impossible to identify the precise metabolic function of individual species in a complex microbial community, we have made the classification according to the predominant (or more likely) metabolic type at the level of genus, informed by the literature and by the type of metabolic products detected in the medium. We have used this approach before in the analysis of the microbial communities in single MFCs (Stratford et al., 2014; Grüning et al., 2015).

**Table 2:** The 14 most abundant genera found in the anodic biofilm and anolyte suspension of triplicate MFC cascades, classified according to their most likely metabolic function.

Genus	Metabolism	Anode biofilm main	Suspension main	Biofilm cascade ↗	Biofilm cascade ↘	Reference
*Anaerotruncus*	F		X		X	Lawson et al., 2004
*Bacteroides*	F	X			X	Nishiyama et al., 2009
*Clostridium*	F		X		X	Holt, 1994
*Comamonas*	R	X		X		Gumaelius et al., 2001
*Dysgonomonas*	F	X				Lawson et al., 2002
*Enterococcus*	F		X			Cai, 1999
*Ethanoligenes*	F		X		X	Xu et al., 2010
*Lactococcus*	F		X		X	Tanaka et al., 2002
*Leuconostoc*	F		X			Giglio and McCleskey, 1953
*Proteiniclasticum*	F		X		X	Zhang et al., 2010
*Rummeliibacillus*	R	X		X		Her and Kim, 2013
*Sporobacterium*	F		X		X	Mechichi et al., 1999
*Stenotrophomonas*	R	X		X		Yu et al., 2009
*Streptococcus*	F		X		X	Thomas and Turner, 1981

**F*, fermentative metabolism; *R*, anaerobic respiration; Anode Biofilm Main: more abundant in anodic biofilm than anolyte suspension; Suspension Main: more abundant in anolyte suspension than in anodic biofilm; Biofilm Cascade↗: increase in abundance in anodic biofilm across MFC cascade; Biofilm Cascade↘: decrease in abundance in anodic biofilm across cascade*.

**FIGURE 6:**
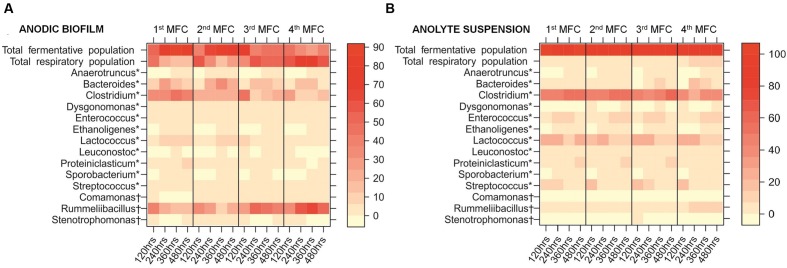
**Heatmap showing the percentage abundance of the 14 most abundant genera within: **(A)** the anodic biofilm of each MFC in the cascade; **(B)** the anolyte suspension of each MFC in the cascade**. Results shown are average percentage abundance of three biological replicates. Total fermentative population represents the total number of fermentative species out of the top 14 most abundant genera. Total respiratory population represents the total number of anaerobic respiratory species out of the top 14 most abundant genera. ^∗^Fermentative metabolism most likely, ^†^Anaerobic respiration most likely.

The shifting dynamics of these 14 genera in the anodic biofilm and the anolyte suspension across the MFC cascade is shown in **Figure 6**. The heatmap shows the temporal changes of the most abundant genera in the individual MFCs, and the changes in abundances according to the position of the relevant MFC in the cascade. While the overall temporal trend along the cascade shows a clear increase of the respirators and a decrease of the fermenters, the time-course of the first two MFCs shows a slight increase of the fermenters, and an even slighter increase of respirators in the anolyte. At 120 h, the system has probably not reached the definitive composition, as it is suggested by the lower power output and the metabolite profile. Previous work has differentiated between electrical steady state and biological steady state (Ren et al., 2011), and our observation could be explained using this concept: even though the system might have reached steady electrical output, the biofilms were still maturing at 120 h, reaching the biological steady state at 240 h. Our results show that the system is fully mature at longer times: The segregation of the communities in the MFC cascade occurs as the biofilm reaches biological steady state.

The % relative abundance of genera with fermentative metabolism in each MFC decreased from an average of 75.6% (±10.1%) of the anodic biofilm in the first MFC in the cascade to just 36.7% (±8.3%) in the final MFC (**Figure 6A**). The largest changes were observed for *Proteiniclasticum* (7.4-fold decrease), *Clostridium* (2.5-fold decrease), and *Anaerotruncus* (1.5-fold decrease). Conversely, the % relative abundance of anaerobic respiring genera in each MFC increased from an average of just 24.4% (±10.1%) of the anodic biofilm in the first MFC in the cascade to 63.3% (±8.3%) in the final MFC (**Figure 6A**). The largest increases were observed for *Comamonas* (7.7-fold), *Rummeliibacillus* (2.4-fold), and *Stenotrophomonas* (twofold).

The composition of the suspended community in the anodic chamber (**Figure 6B**) was very different to that of the biofilm, and remained constant across the cascade. On average, 57.0% (±5.6%) of the anodic biofilm community is composed of a fermentative population, whereas in the anolyte suspension, the fermentative population makes up on average 96.1% (±1.4%) of the community. Fermentative metabolism does not require the use of external EAs (the anode, in an MFC), and metabolic energy is obtained by the conversion of substrates into fermentation products, which are normally excreted. On the other hand, the anaerobic respiratory population cannot fully oxidize those fermentation products without an external EA, and so are in high abundance in the anodic biofilm community.

The most abundant genus found in the MFC cascade is *Clostridium*, a member of the phylum *Firmicutes*. *Firmicutes* have been found to dominate anodic communities of acetate-fed MFCs (Aelterman et al., 2006; Wrighton et al., 2008; Beecroft et al., 2012) and cellulose-fed MFCs (Rismani-Yazdi et al., 2007). In our study, the abundance of *Clostridium* species in the anodic biofilm community (average relative abundance of 24.6±13%, **Figure 6A**) was nearly half of that in the anolyte suspension (46.8±11%, **Figure 6B**). *Clostridium* species are able to ferment glycerol (Yazdani and Gonzalez, 2007), xylose (Balasubramanian et al., 2001), and various pentosans (Rogers and Baecker, 1991; Broda et al., 2000) into acetate.

Similarly to *Clostridium*, species of the fermentative *Lactococcus* genus were abundant in the anolyte suspension (average relative abundance of 18.2±1.8%, **Figure 6B**), but were much less abundant in the anodic community (average relative abundance of 4.7±1.4%, **Figure 6A**). While *Lactococcus* species are able to ferment xylose into lactic acid and acetic acid (Tanaka et al., 2002), *Lactococcus lactis* can transfer electrons to an anode using mediators such as quinones (Freguia et al., 2009) or flavins (Masuda et al., 2010).

The third most abundant fermentative genus in the anodic community was *Bacteroides* (average relative abundance of 13.3±4.6%, **Figure 6A**), which has been previously reported to be abundant in anodic communities (Beecroft et al., 2012; Jia et al., 2013). *Bacteroides* species are able to ferment a range of plant polysaccharides and xylans (Salyers et al., 1977; Cooper et al., 1985) as well as some of the sugars present in DDGS, such as xylose (Turner and Roberton, 1979).

This is the first time a *Rummeliibacillus* species has been shown to be dominant in anodic communities (**Figure 6A**). *Rummeliibacillus*, previously classified as *Bacillus*, are generally unable to ferment common hexoses, pentoses, hexitols, disaccharides, and trisaccharides (Nakamura et al., 2002). *Rummeliibacillus* produces the menaquinones MK-7 and MK-8 (Her and Kim, 2013), which mediate anaerobic respiration by shuttling electrons from an oxidized fermentation product to an EA. Despite playing a dominant role in the anodic biofilm (average relative abundance of 34.5±3.8%), the abundance of *Rummeliibacillus* species is low in the anolyte suspension, with an average relative abundance of 3.6±1.2% (**Figure 6B**).

The effect of the abundance of fermenters in the biofilm across the cascade on the power output was tested by a linear effects model (**Table 1**, Model 3), which showed that the fermenters responsible for converting DDGS components into acetate have a statistically negative correlation with peak power (β = -0.50, *p* ≤ 0.0001). This confirms that along the cascade, the fermentative population in the suspension generates the substrates for the electrogenic population in the biofilm.

## Discussion

Relationships between metabolite pools, microbial functional types and power output were characterized in a hydraulic cascade of MFCs inoculated with a complex microbial community. The results obtained indicate that the microbial community in the anode self-organizes in accordance to the substrate availability. Considered individually, the MFCs showed changes in the composition of the community and in the metabolic profile, attributable to the acclimation and maturation of the community to the available substrates. When considering the whole cascade, the abundance of fermentative genera is highest in the first MFC, and decreases across the cascade. This can be explained by the decrease in fermentable substrates, which prevents the formation of a stable fermentative community in the biofilm. The anaerobic respirators, on the other hand, become more abundant in the anodic community further along the cascade due to the highest availability of SCFAs. Respirator species can oxidize those compounds and utilize the anode as the EA, resulting in the observed trends in power output.

The microbial communities in the anode could also be separated into two groups, one associated to the biofilm and another associated to the anolyte suspension. Those populations were composed by fermentative and anaerobic respiratory genera, as determined by both taxonomic and extracellular metabolite analyses. Mean genera abundances were very different, with the vast majority (>90%) of the anolyte suspension being composed of fermentative microorganisms, whereas in the anodic biofilm community the fermentative population abundance was only 50%. These differences suggest that the anode is selecting for anaerobic respirators with redox systems best suited for utilizing the anode as an EA.

Our experimental data provide evidence that fermentative pathways are active in the MFCs, converting the components of DDGS (carbohydrates and pentosans) to yield free sugars (e.g., glucose, xylose) that can be metabolized to yield the SCFAs commonly used by electrogenic species. This is in agreement with the presence of fermentative metabolism products which accumulated across the MFC cascade, notably acetate (**Figure 3C**), propionate and succinate (Supplementary Information). The increased concentration of acetate in the fourth MFC indicates that the fermentative population in the early stages has converted all fermentable substrates into SCFAs for respiration. These might have been accumulated due to the external resistance of the MFC being too high to drain all acetate electrons through the anode.

The fermentative population themselves contribute little to power production but produce acetate, which can only be completely oxidized by the anaerobic respiratory population if electrons are donated to the anode (Grüning et al., 2015; Sun et al., 2015). Therefore for complex substrates, fermentative species play a crucial role in the ability of microbial communities to fully oxidize carbohydrates in MFCs. Although a pre-fermentation step prior to feeding MFCs could be used to provide substrates for the respiratory population (Sharma and Li, 2010; Goud and Mohan, 2011), our cascade system naturally generates a selective environment for the evolution of communities with the right composition and activities for the given substrate and HRT. Considerably longer residence times would be required in single MFCs, to achieve the same bioconversion of complex substrates to power production and the HRT would need to be fine-tuned to achieve the optimal proportions of fermentative and respiratory communities. From a scalability perspective, studies focusing on improving single MFC performance have demonstrated that increasing anodic chamber size results in an increase in internal resistance (Ieropoulos et al., 2008); connecting a series of smaller, more efficient MFCs in hydraulic cascade overcomes this limitation (Walter et al., 2015). Understanding how MFCs will perform depending on their position in the cascade is an important step toward application of this technology.

To the best of our knowledge, this is the first exhaustive analysis of microbial communities in MFC cascades, showing the link between the composition of the community, the metabolic profile and the power output, and demonstrating how the relationship between the fermentative and anodophilic populations underpin the observed increase in power yields across the cascade. Previous reports have mentioned the existence of syntrophic relationships in single MFCs fed with simple carbon sources (Freguia et al., 2008; Yamamuro et al., 2014), or simplified systems involving only two species (Kimura and Okabe, 2013).

We show here that a cascade of multiple MFCs is an ideal platform for the study of those relationships, as it provides a high-resolution map of the interactions between species. The presence of different anodic communities in the MFCs in the cascade, as demonstrated by the taxonomic analysis and illustrated in the heatmap (**Figure 6**), together with the different metabonomic profiles discussed above (**Figures 3–5**) clearly indicate that the effect of a cascade set up is the segregation of different communities according to the metabolic functions prevailing in each MFC unit. The native initial community is therefore “stretched” along the different MFCs in the cascade, ensuring that the generated sub-communities act optimally. Changes in the structure of the cascade (e.g., changing the number of MFCs and/or the HRT) will promote changes in the structure of the sub-communities, allowing not only for a more efficient performance but also to disentangle the taxonomic structure of functional consortia (Dolfing, 2014). This approach could be extended to the study of other equally complex processes where the concerted activity of microbial communities is required.

## Author Contributions

DH designed and performed the experiments, and contributed in the analysis of the data, discussion and preparation of the manuscript. AS performed the genotypic analysis and contributed to the discussion. SD contributed with the bioinformatic analysis. JVL performed the metabolic analysis and discussion of the data. JS performed the statistical analysis and contributed to the discussion. AG contributed in the interpretation and discussion of the electrochemical results. MB contributed to the design of the experiments, the analysis of the metabolic data and the discussion. JM contributed in the design of the genotypic and metagenomic experimental details, and contributed in the analysis of the results. CAR supervised the design of the experiments, the overall analysis and discussion of the results, and prepared the manuscript.

## Conflict of Interest Statement

The authors declare that the research was conducted in the absence of any commercial or financial relationships that could be construed as a potential conflict of interest.

## Supplementary Material

FIGURE S1 **Performance at 120 h of MFCs inoculated with aerobic and anaerobic enriched microbial community derived from lignocellulose compost**. Lignocellulosic compost was used to inoculate chemostats continuously fed with 10% DDGS medium. The process was performed under aerobic and anaerobic conditions, and both enriched communities were tested for their electrogenic activity in single MFCs. The aerobically enriched community produced higher peak power at 120 h, and was therefore chosen as the inoculum for the MFC cascade.
