# Supplementary figures and images for: Segregation of the Anodic Microbial Communities in a Microbial Fuel Cell Cascade
Source: Front Microbiol. 2016 May 11;7:699. doi: 10.3389/fmicb.2016.00699 (PMC4863660; doi:10.3389/fmicb.2016.00699)

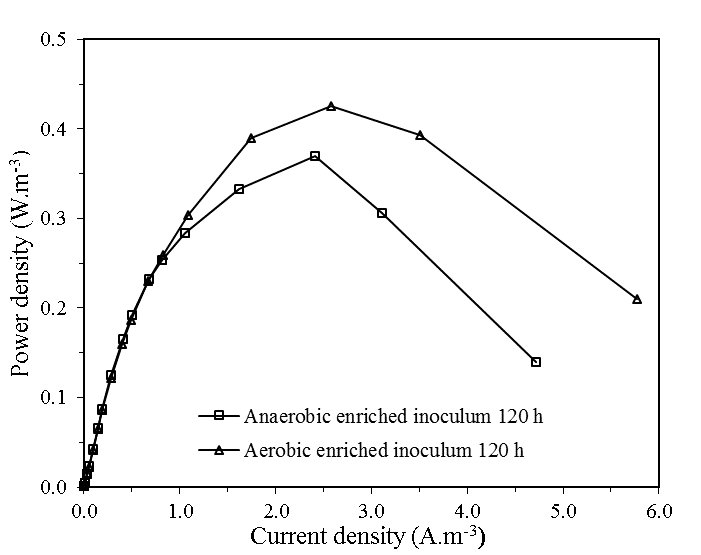

Supplement: FIGURE S1 — Performance at 120 h of MFCs inoculated with aerobic and anaerobic enriched microbial community derived from lignocellulose compost. Lignocellulosic compost was used to inoculate chemostats continuously fed with 10% DDGS medium. The process was performed under aerobic and anaerobic conditions, and both enriched communities were tested for their electrogenic activity in single MFCs. The aerobically enriched community produced higher peak power at 120 h, and was therefore chosen as the inoculum for the MFC cascade. [file Image_1.PNG]
